# Improving Physical Assessment and Clinical Judgment Skills without Increasing Content in a Prelicensure Nursing Health Assessment Course

**DOI:** 10.3390/nursrep11030057

**Published:** 2021-08-02

**Authors:** Kathryn Kinyon, Shannon D’Alton, Kristen Poston, Sarah Navarrete

**Affiliations:** College of Nursing, Medical University of South Carolina, Charleston, SC 29425, USA; vaillan@musc.edu (S.D.); mcdanikf@musc.edu (K.P.); navarres@musc.edu (S.N.)

**Keywords:** physical exam, nursing assessment, prelicensure nurse, health assessment, physical assessment, undergraduate nursing education

## Abstract

One hundred twenty-six assessment skills are taught in prelicensure nursing health assessment courses, yet 30 skills are used on a routine basis in practice. New nurses struggle to apply their acquired knowledge in clinical settings. Method: A literature review was completed. Based on the results, a first semester health assessment course in a southeastern accelerated baccalaureate nursing program was redesigned. Lectures and skills labs were adjusted to focus on the most critical assessment skills. To foster critical thinking and clinical judgement, a health assessment post conference was added where students completed concept maps, system specific case studies, nursing priority setting, and patient teaching plans. Results: Outcome surveys were completed by second semester faculty. Prior to course adjustments, 33 percent of students did not meet the benchmark. Following course changes, all students met or exceeded the benchmark. Conclusion: Focusing on critical assessment skills will increase student nurses’ ability to deliver safe patient care.

## 1. Introduction

Patients depend on a nurse’s ability to recognize and respond quickly to changes in their condition. Clinical judgement involves the process of clinical thinking, clinical reasoning leading to a clinical judgement. This process requires a nurse to recognize patient cues, and critically analyze the data. This is followed by making appropriate decisions to optimize patient outcomes [1,2,3,4]. Yet, studies document that new nurses struggle to apply their recently acquired knowledge in the rapid-paced, ever-changing clinical setting [5]. In one survey, 77 percent of new nurse graduates failed to demonstrate clinical competency [1]. In another study, 65 to 76 percent of inexperienced RNs were unable to meet entry level clinical judgment skills, and the majority had difficulty translating knowledge and theory into practice [6]. This should not be surprising as new graduate nurses are likely to have theoretical competence but lack practical proficiency. In addition, new nurses often use concrete thinking and depend on technology to evaluate a patient’s condition, thus missing the clues which highlight a bigger picture [5]. The implementing of clinical judgement is made more difficult due to the ever-increasing amount of information nurses must absorb. It has been estimated that nursing knowledge doubles every six years [1]. The purpose of this paper is to present how a Bachelor of Science Nursing Health assessment course was redesigned to improve health assessment skills competencies without increasing course content.

In a National Council of State Boards of Nursing survey, employers reported concern regarding new graduate nurses’ readiness to enter practice. Fewer than 50 percent of employers reported “yes definitely” when asked if new graduates were ready to provide safe and effective care [7]. Other studies supported this assessment, indicating that only 23 percent of novice nurses were well prepared for clinical practice [8]. Supporting the concern for nursing preparedness, the World Health Organization stated that medication errors were the 14th leading cause of death globally and the 3rd leading cause of death in the United States [7]. Lack of readiness can significantly contribute to preventable errors [9].

The foundation for developing nursing interventions starts with clinical competency. However, it has been argued that the comprehensive physical exam has become a “sacred cow” in nursing education [8]. Some have asked, are we trying to teach too much. To answer this question, Giddens and Eddy (2009) [10] conducted a survey which found that more than 50 percent of nursing schools taught 122 specific physical assessment skills. Other researchers who surveyed baccalaureate nursing programs found that over 126 physical assessment skills were being taught [6,10,11,12,13,14,15,16,17,18,19].

In 2007, Giddens [16] published a landmark study to determine the physical assessment skills routinely used by the bedside nurse. It was found that of the 126 physical exam techniques taught, only 30 were used either daily or weekly in the nurses’ clinical practice. Additional studies replicating Giddens’ work, comparing the number of critical nursing assessment skills being taught to what practicing nurses routinely preform, found that out of 126 assessment skills taught, only about 30 were used on a daily or weekly basis [6,10,11,12,13,14,15,16,17,18,19]. Giddens (2007) [16] concluded that nurse educators should reconsider which assessment skills are needed for entry into practice.

In 2017, Kohtz, et al. [12] published a study which replicated Giddens’ original 2007 study by surveying fourth semester students. The results determined the frequency of physical exam skills used in the students’ clinical care. Of the 126 physical assessment skills surveyed, only 21 skills were used each time the student was in clinical practice. An additional nine physical exam skills were performed two to five times per week. In another study evaluating final semester nursing students, researchers found that of 126 skills taught, 53 skills were never performed during the students’ clinical practice [10]. Perceived barriers to utilizing more advanced assessment skills included a lack of confidence, doubt on the impact on patient care, and lack of nursing role models [10]. Anderson, et al. (2014) [11] surveyed 900 nurses using Gidden’s 126 skills and asked the nurses to report the frequency of use. Based on survey, only 37 competencies were felt to be essential components of the physical assessment [11].

As new prelicensure nursing health assessment faculty and experienced nurse practitioners, course faculty wondered why we were teaching advanced practice physical assessment skills to first semester nursing students. These skills are rarely, if ever, used in general practice by a registered nurse. Examples of rarely used physical assessment skills included: diaphragmatic excursion, liver span, chest percussion and assessment of cardiac borders. In addition, course faculty were hearing from second semester medical/surgical clinical faculty that students lacked confident in the basic patient physical assessment skills. Clinical faculty noted that students were struggling to apply physical exam findings in a meaningful way.

Poje (2020) [20] wrote of a similar experience when discussing a patient’s exam findings with a student. The student had been taught many exam techniques but had difficulty determining the importance of each finding. Just teaching assessment skills alone does not teach a nurse how to analyze the exam findings. This lack of clinical judgement application can increase the risk of missing the signs of patient deterioration [19]. To graduate students who can apply clinical judgement to provide safe, effective care, faculty must discern the “need to know” from the “nice to know”.

Given the need to improve clinical judgement while not increasing content, it is time to reexamine teaching assessment skills which are infrequently or rarely used by a registered nurse [10]. Faculty must look for ways to improve student preparation for practice. However, increasing the content-laden curriculum will only exacerbate information overload. This leaves faculty struggling to balance what is critical to know without overwhelming their students.

With the emphasis on clinical reasoning and clinical judgement, it was time to rethink how health assessment was being taught. Since there was going to be a change in textbooks, it seemed the perfect time to redesign the course. The purpose of this paper is to present how a Bachelor of Science Nursing Health assessment course was redesigned to improve health assessment skills competencies without increasing course content.

## 2. Materials and Methods

**Figure 1:**
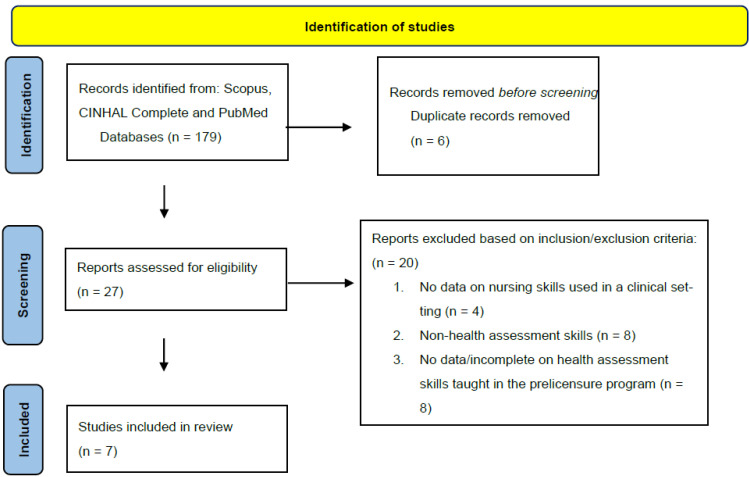
Literature Review.

The Medical University of South Carolina Institutional Review Board (MUSC-IRB) determined this project a quality improvement/program evaluation project, thus MUSC-IRB review was not required. Patient consent was waived due to the project being deemed a quality improvement/program evaluation project. A comprehensive literature review was completed. Key search words used in the literature search were physical exam, nursing assessment, prelicensure nurse, health assessment, physical assessment, and undergraduate nursing education. In December of 2019, Scopus, CINHAL Complete, and PubMed databases were searched for research published in the previous five years. Inclusion criteria included: prelicensure nursing program, health assessment curriculum with a focus on a comprehensive list of health assessment skills, nursing skills actively used in clinical care, and English speaking. Exclusion criteria included: non-health assessment curriculum, advanced nursing programs, no data on nursing health assessment skills taught or no data on nursing health assessment skills used in a clinical setting, and non-English speaking programs. Initial results yielded 179 articles. However, several landmark studies were outside the timeline but were included in the final screening. Twenty-seven articles were screened with seven meeting inclusion criteria (Figure 1).

The articles which met inclusion criteria were reviewed and data highlighting critical nursing assessment skills summarized (Table 1). Data were then assessed to determine if there was consensus among the research on critical physical assessment skills. Based on the review, consensus across studies was found as to which assessment skills were critical for a nurse to perform competently upon entry into practice.

Course faculty met to review the literature results and to compare skills currently being taught with the critical competency skills. Prior to the curriculum redesign, students were required to learn 195 assessment techniques. Required skills were decreased from 195 to 75 (a decrease of 62 percent). Areas which saw the greatest reduction in required skills were: head, eyes, ears, and neck and throat (HEENT), decreasing from 47 separate skills to 11 and neurological exam from 57 skills to 24 (Table 2). Specific examples of skills eliminated included: chest percussion, chest excursion, JVP measurement, liver hooking, percussing liver borders, thyroid exam, and otoscopic exam.

Faculty redesigned the health assessment lab and final physical assessment check-off to focus on the identified critical skills necessary to complete a head-to-toe assessment competently. Lectures were adjusted to focus on the critical assessment skills. A one-hour post-lab conference was initiated to introduce the use of clinical judgement when completing a health assessment. During the post-conference, students completed a concept map on a disease appropriate to the system being studied and then applied the information to an original case study. The case study required students to identify cues in the patient health history. Students then determined what type of physical exam techniques should be applied when examining the patient. Finally, students were asked to determine the patient’s first, second, and third nursing priorities and develop a teaching plan.

Prior to redesigning the course, students were only tested on two systems which were randomly assigned. In the redesigned course, students were required to complete a full head-to-toe assessment to demonstrate competencies in all the critical assessment skills.

The initial pre-course change surveys were sent out to the medical/surgical clinical faculty at the start of spring semester in January of 2020. Clinical faculty were asked to evaluate second semester students who had just completed the health assessment course. Students were evaluated on their ability to perform a comprehensive physical assessment based on the criteria of “does not meet expectations”, “meets expectation”, or “exceeds expectation”.

The health assessment curriculum changes were implemented at the beginning of the spring semester in January of 2020. In March of 2020, due to COVID-19, students were no longer allowed on campus, and all course and skills labs were moved online. Due to COVID-19 restrictions, students submitted a video of themselves completing a comprehensive physical exam, and the videos were reviewed and graded by their lab faculty. Due to the abrupt change to online learning, a post-curriculum change survey was not sent out after the spring semester.

In the fall of 2020, face-to-face skills labs were restarted. At the end of the fall semester, students demonstrated their ability to complete a comprehensive physical exam competently with faculty present in real time. Post-curriculum change surveys were sent out to the clinical faculty in January of 2021. In December of 2021, the first cohort who completed the revised health assessment curriculum will graduate and take their licensing exam.

## 3. Results

The results of the pre- and post-course change surveys showed significant improvement in student outcomes. Prior to course revisions, 30 percent of students did not meet expectations followed by 50 percent meeting expectations and 20 percent exceeding expectations. Post-course redesign surveys demonstrated a shift to 50 percent meeting expectations and 50 percent exceeding expectations.

By decreasing the number of skills students were required to be proficient in, students were able to hone their skills on the important techniques and learn to apply clinical judgement to exam findings. This positive result was supported by clinical faculty who noted improvement in the students’ ability to assess their findings and begin to apply the critical thinking necessary implement nursing care plans. Students voiced increased confidence in their ability to perform a head-to-toe assessment on the end of course evaluation surveys.

## 4. Discussion

The COVID-19 pandemic created changes in our healthcare delivery models through the increased use of telehealth and expansion of the nurses’ scope of practice. However, the complexity of patient care has only increased and thus increased the risk for adverse outcomes. Studies indicate novice nurses tend to think in a concrete/linear process. They struggle to process large amounts of complex information and anticipate changes in a patient’s situation [15]. This leads to difficulty determining which clinical situations need immediate attention and which are less acute.

To intervene quickly and prevent poor patient outcomes, nurses must be able to recognize both the overt and subtle cues when performing a patient assessment [13]. If one lacks the ability to apply clinical judgement, there is an increased risk of missing the signs of patient deterioration [10]. Student nurses need to start shifting from concrete thinking to an analytical approach to patient care and assessment. They need to recognize a situation in which a particular aspect of theoretical knowledge applies and begin to develop practical knowledge that allows refinement, extension, and adjustment of textbook knowledge [6]. This can be accomplished by developing confidence in performing basic assessment skills and becoming competent in translating the findings into appropriate nursing actions [8].

Our revised approach to a nursing health assessment course is unique given the strong emphasis on critical thinking and clinical judgement without an increase in content. Sound clinical judgment includes multiple components all intertwined together. The nurse must understand the pathophysiology and diagnostic aspects of a patient’s clinical signs/symptoms and disease process. The nurse must be able to assess the illness experience for both the patient and family and evaluate their physical, social, emotional strengths and coping resources [11]. With the reduction in required skills for which a student must demonstrate competency, students had time to focus on the clinical aspects of the exam and correlate it to the patient disease process. Using a nursing concept map to focus on a specific disease process enabled students to synthesize and apply the information to specific health assessment skills. Scaffold learning occurs as students begin to understand basic disease processes and then apply the information to patient case students. Finally, utilizing this process helps students begin to practice the implementation of patient-centered care.

It is time to re-examine those skills that are used infrequently or rarely and to discuss with our colleagues the usefulness of teaching these skills. This is not to advocate that all skills used infrequently or rarely should be abandoned, only that each skill should be thoughtfully considered and discussed. Douglas et al., (2016) [17] proposed a “Core + Cluster” approach to nursing physical assessment. This approach would include the 37 essential skills routinely performed with the addition of cluster skills for specialized areas. This approach defines how our faculty determined the critical skills students needed to learn. Students were still required to learn all four components of a physical exam (inspection, palpation, percussion, and auscultation). However, additional skills were added based on their importance in determining changes in a patient’s health status. Thus, certain neurological and respiratory assessment skills were still included in the final assessment even though they were not part of the 37 essential skills.

Evaluation of this quality improvement project is ongoing. In December of 2021, the first cohort will complete the program under the revised curriculum. Adjustments may be made after the licensure board results are reviewed. Based on the previous semester experiences, several revisions will be implemented in the Fall 2021 semester which will include completion of an empathy concept map which focuses on skills to improve wholistic care and gender inclusive interview skills, and enhanced role playing to improve comprehensive interview skills.

## 5. Conclusions

The Institute of Medicine recommended transforming nursing education to close the practice-education gap [11]. Given the increased emphasis on improving graduate nurses’ readiness to enter practice, everything should be on the table for review and revision including physical assessment skills. Faculty must look for ways to improve student preparation for practice. However, increasing the content-laden curriculum will only exacerbate information overload. This leaves faculty struggling to balance what is critical to know without overwhelming their students. One option is to re-examine closely which assessment skills are being taught and consider eliminating skills which are infrequently or rarely used by a registered nurse [10]. Focusing on critical physical assessment skills, which include application of clinical judgement skills in real-life scenarios, will increase student nurses’ ability to observe, assess, and prioritize patient concerns, leading to an improved ability to deliver safe client care.

## Figures and Tables

**Table 1:** Essential Skills Summary Table.

Essential Skills	Anderson et al. (2014) [11]	Kohtz, et al. (2017) [12]	Giddons, (2007) [16]	Douglas, et al. (2016) [17]	Egilsdottir, et al. (2019) [15]	Birks, et al. (2014) [18]	Secrest et al. (2005) [14]
*Vital Signs*							
Assess pain				X			
BP				X	X		
RR				X	X		
HR				X	X		X
Sa02				X	X		
Body temp				X			
Weight				X			
Height				X			
*Integumentary*							
Inspect skin color	X	X	X	X		X	X
Skin lesions	X	X	X	X		X	X
Skin wounds	X	X	X	X		X	X
Estimate skin fold					X		
*Head*:							
Inspect facial structures	X	X				X	X
Oral cavity	X	X	X	X	X		
*Ears*:							
Assess hearing based on conversation	X	X	X		X	X	
*Eyes*:							
Inspect external eyes	X	X	X	X	X	X	
Visual acuity	X	X			X	X	
PERRLA	X	X	X	X	X	X	
*Thorax*:							
Inspect chest shape	X	X	X		X	X	X
Breathing effort	X	X	X		X	X	
Palpate chest wall expansion	X				X	X	
Auscultate breath sounds	X	X	X	X	X	X	X
Ability to cough							
Percuss lungs				X	X	X	
*Abdomen*:							
Inspect abdomen	X	X	X	X	X	X	X
Auscultate bowel sounds	X	X	X	X	X	X	X
Palpate for tenderness/distention	X	X	X	X	X	X	X
Assess stool			X	X	X	X	
Percuss abdomen							
*Cardiovascular*:							
Inspect cap refill	X	X	X	X	X	X	X
Palpate distal pulses	X	X	X	X	X	X	X
Inspect/palpate edema	X	X	X	X	X	X	X
Palpate extremities temp	X	X	X	X		X	X
Inspect extremities for color and hair growth	X	X	X	X	X	X	X
Auscultate carotid artery	X					X	
Inspect jugular pulsation	X	X	X	X	X	X	X
Auscultate heart sounds	X						
*Musculoskeletal*:							
Inspect back/Spine	X		X			X	
Inspect muscles/extremities size/symmetry	X		X			X	X
Palpate extremities/joints/calve for tenderness	X	X	X	X		X	
Observe ROM	X	X	X	X		X	X
Assess muscle strength	X	X	X	X	X	X	X
*Neurological*:							
Assess mental/LOC	X	X	X	X		X	
Glascow Coma Scale	X		X			X	
Evaluate speech	X	X	X	X		X	
Facial movement/sensation	X	X	X		X	X	
Assess gait							
CN I-XII	X	X	X	X	X	X	
Patella/Planter reflex					X		
Gag reflex					X	X	
Total Skills	34	28	30	32	30	36	19

**Table 2:** Physical Assessment Skills Percent Reduction.

Skills	HEENT	Cardiovascular	Thorax	Neurological	Abdomen	Musculoskeletal
Decreased by	74%	39%	30%	58%	73%	74%
